# Efficient formation of a massive quiescent galaxy at redshift 4.9

**DOI:** 10.1038/s41550-024-02424-3

**Published:** 2024-11-28

**Authors:** Anna de Graaff, David J. Setton, Gabriel Brammer, Sam Cutler, Katherine A. Suess, Ivo Labbé, Joel Leja, Andrea Weibel, Michael V. Maseda, Katherine E. Whitaker, Rachel Bezanson, Leindert A. Boogaard, Nikko J. Cleri, Gabriella De Lucia, Marijn Franx, Jenny E. Greene, Michaela Hirschmann, Jorryt Matthee, Ian McConachie, Rohan P. Naidu, Pascal A. Oesch, Sedona H. Price, Hans-Walter Rix, Francesco Valentino, Bingjie Wang, Christina C. Williams

**Affiliations:** 1 Max Planck Institute for Astronomy, Heidelberg, Germany; 2 Department of Astrophysical Sciences, Princeton University, Princeton, NJ USA; 3 Cosmic Dawn Center, University of Copenhagen, Copenhagen, Denmark; 4 Department of Astronomy, University of Massachusetts, Amherst, MA USA; 5 Kavli Institute for Particle Astrophysics and Cosmology and Department of Physics, Stanford University, Stanford, CA USA; 6 Centre for Astrophysics and Supercomputing, Swinburne University of Technology, Melbourne, Victoria Australia; 7 Department of Astronomy & Astrophysics, The Pennsylvania State University, University Park, PA USA; 8 Department of Astronomy, University of Geneva, Versoix, Switzerland; 9 Department of Astronomy, University of Wisconsin–Madison, Madison, WI USA; 10 Department of Physics and Astronomy and PITT PACC, University of Pittsburgh, Pittsburgh, PA USA; 11 Department of Physics and Astronomy, Texas A&M University, College Station, TX USA; 12 George P. and Cynthia Woods Mitchell Institute for Fundamental Physics and Astronomy, Texas A&M University, College Station, TX USA; 13 INAF - Astronomical Observatory of Trieste, Trieste, Italy; 14 Leiden Observatory, Leiden University, Leiden, The Netherlands; 15 Institute for Physics, GalSpec laboratory, EPFL, Observatory of Sauverny, Versoix, Switzerland; 16 Institute of Science and Technology Austria (ISTA), Klosterneuburg, Austria; 17 Department of Physics & Astronomy, University of California Riverside, Riverside, CA USA; 18 MIT Kavli Institute for Astrophysics and Space Research, Cambridge, MA USA; 19 European Southern Observatory, Garching, Germany; 20 NSF’s National Optical-Infrared Astronomy Research Laboratory, Tucson, AZ USA

**Keywords:** Galaxies and clusters, Early universe

## Abstract

Within the established framework of structure formation, galaxies start as systems of low stellar mass and gradually grow into far more massive galaxies. The existence of massive galaxies in the first billion years of the Universe, as suggested by recent observations, seems to challenge this model, as such galaxies would require highly efficient conversion of baryons into stars. An even greater challenge in this epoch is the existence of massive galaxies that have already ceased forming stars. However, robust detections of early massive quiescent galaxies have been challenging due to the coarse wavelength sampling of photometric surveys. Here we report the spectroscopic confirmation with the James Webb Space Telescope of the quiescent galaxy RUBIES-EGS-QG-1 at redshift *z* = 4.90, 1.2 billion years after the Big Bang. Deep stellar absorption features in the spectrum reveal that the stellar mass of the galaxy of 10^11^ *M*_⊙_ formed in a short 200 Myr burst of star formation, after which star formation activity dropped rapidly and persistently. According to current galaxy formation models, systems with such rapid stellar mass growth and early quenching are too rare to plausibly occur in the small area probed spectroscopically with JWST. Instead, the discovery of RUBIES-EGS-QG-1 implies that early massive quiescent galaxies can be quenched earlier or exhaust gas available for star formation more efficiently than assumed at present.

## Abstract (web summary)

RUBIES-EGS-QG-1 is an exceptionally massive and mature galaxy discovered just 1.2 billion years after the Big Bang. Its stars formed in an extremely rapid burst, posing a major challenge to all current theoretical models.

## Main

Recent observations with the James Webb Space Telescope (JWST) have unveiled a surprising number of massive galaxies in the early Universe, reaching stellar masses of up to 10^11^ *M*_⊙_ only 800 Myr after the Big Bang^1^. The rapid growth of these massive systems implies a very high conversion efficiency of baryons into stars, which poses a challenge to theoretical models of galaxy formation and possibly even strains the standard cosmological model^2–4^. Even more puzzling is that many of these massive galaxies seem to be quiescent^5–7^, whereas most galaxies in this early epoch have high star formation activity^8^. However, to date, few high-redshift (*z* > 4) massive quiescent galaxies have been confirmed spectroscopically, leaving open major questions regarding their nature and their formation.

**Fig. 1:**
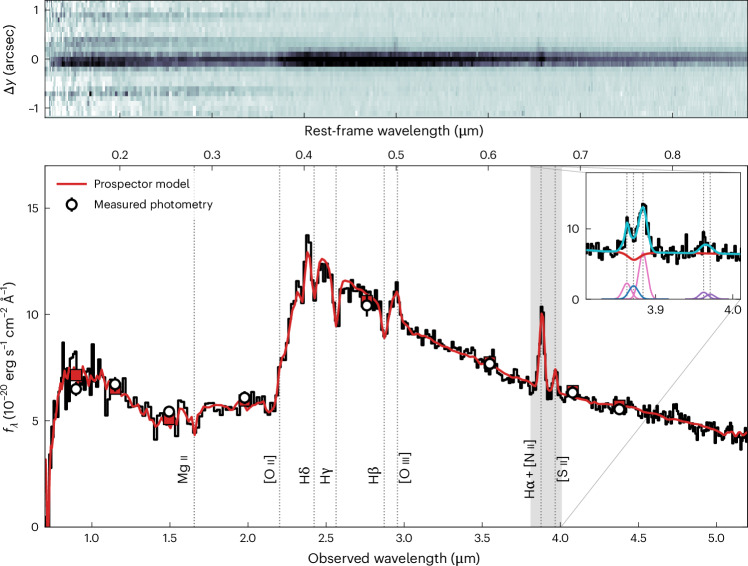
JWST/NIRSpec PRISM spectrum of the massive quiescent galaxy RUBIES-EGS-QG-1 at a redshift of *z* = 4.8976. The 1D spectrum (flux density *f*_λ_; bottom panel) shows deep Balmer absorption lines, like those of post-starburst galaxies at lower redshifts, which implies a lack of star formation in its recent history. Inset, medium-resolution (NIRSpec G395M) spectrum around the wavelength of Hα. Both spectra were calibrated to the measured photometry (error bars on the photometric data points reflect 1*σ* measurement uncertainties) using Prospector. The presence of the emission-line doublets [O iii], [N ii] and [S ii] and the minimal inferred infilling of the Hα absorption line are consistent with AGN activity. The 2D spectrum (top panel) shows the spatial distribution of the emission in the cross-dispersion direction (Δ*y*). In addition to the compact massive quiescent galaxy, we identify the [O III] and Hα emission of a faint companion source that is offset by approximately +0.2 arcsec and -600 km s^−1^ (rest-frame velocity).

RUBIES-EGS-QG-1 was identified in the extended Groth strip (EGS) as a candidate massive quiescent galaxy at *z* > 4.5 based on its red colour, as measured from photometry across 1–5 μm obtained with the near-infrared camera (NIRCam) onboard JWST^9–11^. The source was subsequently selected as a target for a spectroscopic follow-up with the near-infrared spectrograph (NIRSpec) also onboard JWST because of its red colour, F150W − F444W = 2.35, and bright apparent magnitude at long wavelengths, F444W = 22.5. The low-resolution PRISM spectrum of RUBIES-EGS-QG-1 obtained with JWST/NIRSpec (Fig. 1) reveals deep Balmer absorption lines and a strong spectral break at a rest-frame wavelength of 4,000 Å, indicating a lack of star formation in its recent history.

We detected the [O iii]_*λλ*4960,5008_, [S ii]_*λλ*6718,6733_ and blended Hα and [N ii]_*λλ*6549,6585_ emission lines in the PRISM spectrum. A grating spectrum of RUBIES-EGS-QG-1 with higher spectral resolution across 2.9–5.2 μm revealed weak Hα emission infilling the stellar absorption feature and strong [N ii] emission at a redshift of $z=4.8976_{-0.0010}^{+0.0006}$ (Methods). A marginal detection of Hα implies log([N ii]_*λ*6585_/Hα) ≈ 0.5 and exceeds the ratio that can be explained by photoionization from massive stars by a factor of 3 (ref. ^12^). Therefore, the emission lines do not seem to be connected to continuing star formation activity but rather suggest the presence of an active galactic nucleus (AGN), although we cannot rule out the presence of shocked gas^13^. Despite evidence for an AGN, the deep Balmer lines, indicative of a post-starburst system^14^, suggest that the continuum emission of the spectrum is dominated by an evolved stellar population.

The high redshift of RUBIES-EGS-QG-1 allows for stringent constraints on its star formation history in the first billion years of the Universe. To measure the star formation history, we used Prospector^15^ to jointly fit a 21-parameter model to the PRISM spectrum and the observed photometry from the Hubble Space Telescope and JWST (see Methods for a complete description). In brief, the model star formation history was parameterized as 14 time bins of constant star formation, with a common metallicity, dust attenuation, intrinsic velocity dispersion and stellar initial mass function. We fitted nebular emission lines using a simple model in which lines are approximated by Gaussian profiles, but no assumption was made about the origin of the emission. We used a sixth-order polynomial to account for uncertainty in the flux calibration of the spectrum. We explore the effect of deviations in choices from our fiducial model in Methods.

The median model of the sampled posterior is shown in red in Fig. 1. We found a high stellar mass of $9.9_{-0.5}^{+0.4}\times 1{0}^{10}\,{{{M}}}_{\odot }$ and a low star formation rate in the past 100 Myr, ${{\rm{SFR}}}_{100}=4.0_{-1.0}^{+3.5}$ *M*_⊙_ yr^−1^. Together, these correspond to a low specific star formation rate of ${\rm{sSFR}}=4.0_{-0.8}^{+1.0}\times {10}^{-11}\,{{\rm{yr}}}^{-1}$, cementing RUBIES-EGS-QG-1 as the highest-redshift spectroscopic confirmation of a massive quiescent galaxy to date. We inferred low attenuation by dust, with the V-band attenuation from our modelling ${A}_{{\rm{v}}}=0.17_{-0.05}^{+0.06}$. The spectrum unambiguously demonstrates the red rest-frame optical colour is dominated by old stars rather than dust-obscured star formation. This was corroborated by a non-detection in observations by the Northern Extended Millimeter Array (NOEMA) at 1.1 mm, which implies a 3*σ* upper limit on the dust-obscured star formation rate of 120 *M*_⊙_ yr^−1^.

**Fig. 2:**
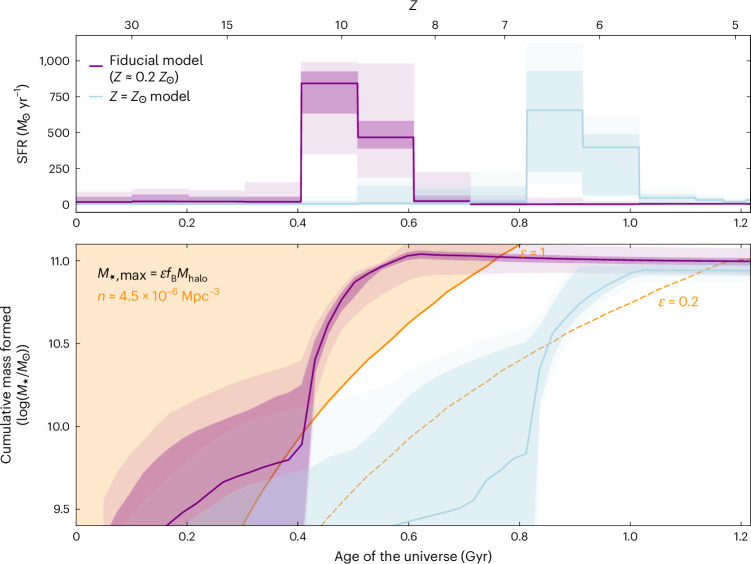
History of stellar mass growth in RUBIES-EGS-QG-1. Top, Star formation history inferred from the modelling to the PRISM spectrum and photometry for the fiducial (free-metallicity) model (purple) and the fixed-solar-metallicity model (blue). Dark (light) shaded regions indicate the 1*σ* (2*σ*) confidence intervals of the posterior distributions. Bottom, Cumulative mass history inferred from the star formation history of the two models. Marked in orange is the maximum stellar mass (M_*,max_) formed for the halo mass (*M*_halo_) of a typical halo at the observed number density of massive quiescent galaxies at *z* > 4 (ref. ^9^), assuming a universal baryon-to-total matter ratio (*f*_B_) and different baryon-to-stellar conversion factors (*ε*). This indicates that a short burst (~300 Myr) of star formation with high efficiency of *ε* > 0.2 is required to form RUBIES-EGS-QG-1, corresponding to an efficiency at or greater than the peak of the stellar–halo mass relation^29^.

We show the resulting star formation history of the galaxy in the top panel of Fig. 2 in purple. RUBIES-EGS-QG-1 assembled its stellar mass within a short burst of star formation, $\Delta t=180_{-10}^{+170}$ Myr, which peaked at a star formation rate ${{\rm{SFR}}}_{{\rm{peak}}}=870_{-140}^{+70}\,{{{M}}}_{\odot }\,{{\rm{yr}}}^{-1}$. For our fiducial model, half of the stellar mass was formed in the first ${t}_{{\rm{form}}}=480_{-10}^{+30}$ Myr of cosmic time, which would mark it as one of the earliest-forming massive galaxies observed. We measured a corresponding quenching timescale ${t}_{90}-{t}_{{\rm{form}}}=100_{-10}^{+10}$ Myr (where *t*_90_ is the time at which 90% of stellar mass formed), indicating that the decline in star formation from its peak in RUBIES-EGS-QG-1 was extremely rapid.

Crucially, we found that the inferred stellar mass, the duration of the star formation burst and the lack of recent star formation activity were largely robust against choices made in the model parameterization and star formation history priors. A full description of models tested can be found in Methods. However, we found that the age of the stellar population depended strongly on the assumed metallicity of the system. Our fiducial fit with the metallicity as a free parameter indicates a low stellar metallicity (~0.2 *Z*_⊙_) and an old stellar population that formed as early as ${z}_{{\rm{form}}}=10.0_{-0.2}^{+0.4}$ and stopped growing by *z* ≈ 8.6. Recent work has shown that the elemental abundance patterns in high-redshift quiescent galaxies differ from the solar abundance patterns typically used in stellar population modelling, which can lead to incorrectly inferred stellar metallicities^16^. When we instead fixed the metallicity of the stellar population to the solar value, we inferred a substantially younger population with a formation redshift of ${z}_{{\rm{form}}}=6.3_{-0.2}^{+0.1}$ as well as more recent quenching (shown as the blue curve in Fig. 2). Although we could not robustly differentiate between these two star formation histories with our current data, we stress that the stellar mass, the timescale and peak of the burst of star formation, and the quenching timescale do not depend significantly on metallicity.

We estimated the dynamical mass of the system using the observed widths of the emission lines and the half-light radius measured from the F444W image, *r*_e_ = 0.55 ± 0.01 kpc (rest-frame wavelength of 0.75 μm; Methods). We found that ${{{M}}}_{{\rm{dyn}}}=2.7_{-0.8}^{+0.7}\times 1{0}^{11}\,{{{M}}}_{\odot }$ is consistent with RUBIES-EGS-QG-1 being a very massive galaxy. The dynamical mass is a factor 3 higher than the stellar mass estimate, although note that the estimated dynamical mass may be elevated by a factor of 2–3 due to non-gravitational motions of the ionized gas that we have not accounted for. The centres of high-redshift massive galaxies are expected to be dominated by stellar mass within the effective radius^17^, which suggests that the stellar mass was not substantially under- or overestimated, despite uncertainties in the initial mass function assumed in our modelling.

The stellar mass surface density within the estimated half-light radius of ${{{\varSigma }}}_{* }( {<} {r}_{{\rm{e}}})=5.{2}_{-0.3}^{+0.2}\times 1{0}^{10}\,{{{M}}}_{\odot }\,{{\rm{kpc}}}^{-2}$ is high but well within theoretical limits for the maximum surface density achievable in a short burst of star formation^18,19^. It is also in line with previous work at *z* < 3, which demonstrated a strong link between low specific star formation rates and high central mass densities, with a threshold density for quiescence that increases toward higher redshift^20,21^. This connection between galaxy structure and the star formation history has been interpreted as a compaction event followed by quenching due to feedback from intense star formation and an AGN^22^, and it is consistent with the high [N ii]/Hα ratio observed in RUBIES-EGS-QG-1, which is indicative of an AGN or shocked gas.

With a peak star formation rate of ~870 *M*_⊙_ yr^−1^ at *z* > 6, RUBIES-EGS-QG-1 had a higher star formation activity than a large sample of the 40 most ultraviolet-luminous sources at *z* ≈ 7 discovered over an area of 7 degrees squared (ref. ^23^). Interestingly, the star formation rate and its star formation rate surface density (*Σ*_SFR_ ≈ 450 *M*_⊙_ yr^−1^ kpc^−2^) are like those of the dusty star-forming galaxy G09 83808 at *z* ≈ 6, which resembles a local ultra-luminous infrared galaxy^24^, and the submillimetre galaxy SPT0311-58 at *z* = 6.9 (ref. ^25^). The star formation history of RUBIES-EGS-QG-1 also matches well with the inferred star formation histories of the brightest red sources found with JWST^26^ at *z* ≈ 7, provided that the light emitted by these sources originates from stars. These different observations suggest that the burst of star formation that formed RUBIES-EGS-QG-1 could too have been strongly obscured by dust. Such a link has also been suggested for the other spectroscopically confirmed massive quiescent galaxy at *z* > 4.5 (ref. ^7^), observed at *z* = 4.658 with a stellar mass of ~3.8 × 10^10^ *M*_⊙_ that formed at *z*_form_ = 6.9 ± 0.2. In comparison with this source, however, RUBIES-EGS-QG-1 stands out for being more than twice as massive. On the other hand, a dust-obscured period of star formation may be difficult to reconcile with the inferred low metallicity and that to date no submillimetre galaxy has been found at *z* > 8 with a star formation rate >200 *M*_⊙_ yr^−1^ (refs. ^23,27^). Therefore, this could, instead, imply that RUBIES-EGS-QG-1 is an exceptionally rare source or that its star formation history is either considerably more extended or more bursty than inferred from our modelling. In the latter scenario, finding ultraviolet-luminous progenitors at *z* ≈ 10 may be difficult, as the probability of discovery depends on both the low number density of extremely massive high-redshift galaxies and on the duty cycle of star formation.

The mere existence of RUBIES-EGS-QG-1 provides an essential constraint on the growth of the most massive galaxies in the early Universe. The bottom panel of Fig. 2 shows the cumulative mass assembly history of the galaxy derived from the modelled star formation history, after subtracting mass returned to the interstellar medium through stellar evolution, as a function of the age of the Universe. The small area targeted with JWST spectroscopy in the RUBIES survey (~100 arcmin^2^ thus far) implies an observed number density of *n* ≈ 3 × 10^−6^ Mpc^−3^ at 4.5 < *z* < 5.5. Based on the photometric selection of candidate massive quiescent galaxies in JWST imaging^9^, the comoving number density of quiescent galaxies of similar mass to RUBIES-EGS-QG-1 (M_*_ > 10^11^ *M*_⊙_) is approximately equally low, *n* ≈ 4.5 × 10^−6^ Mpc^−3^ at 4 < *z* < 5. Hence, we used this number density to estimate the typical dark matter halo mass (*M*_halo_) at a fixed redshift and derive an approximate limit on the total baryonic mass available within the halo^3^. This was then converted to a maximum stellar mass at a given redshift by assuming M_*_(*z*) = *εf*_B_*M*_halo_(*z*), where *f*_B_ is the cosmic baryon fraction (15.6%)^28^ and *ε* is the baryon-to-star conversion efficiency. We plotted this expected stellar mass for two values of the baryon efficiency: *ε* = 1 (assuming the total conversion of baryons to stars) and the efficiency at the peak of the stellar–halo mass relation of *ε* = 0.2 (ref. ^29^). The cumulative mass assembly history of RUBIES-EGS-QG-1 implies a high efficiency of star formation of *ε* > 0.2. The low-metallicity model even suggests that the galaxy was converting baryons to stars with near-perfect efficiency.

Neither the extremely rapid mass assembly nor the early quenching of RUBIES-EGS-QG-1 are consistent with predictions from large-volume (200^3^ to 800^3^ Mpc^3^) cosmological hydrodynamical (FLARES^30^, Magneticum Pathfinder^31^ and TNG300^32^) and semi-analytic (GAEA^33^ and SHARK^34^) simulations of galaxy formation. Although some of these models can produce galaxies that are quiescent at *z* ≈ 4.5–5.0 and are as massive as RUBIES-EGS-QG-1, such systems are extremely rare. The comoving number densities were approximately 1 to 10 × 10^−8^ Mpc^−3^ in the different simulations and correspond to very massive haloes. In comparison with the estimated number density of RUBIES-EGS-QG-1, this implies a probability of 1% (2*σ* outlier) that such a source is observed in the small area probed spectroscopically with JWST (~100 arcmin^2^ for the RUBIES programme). However, at higher redshifts, the comoving number density of quiescent galaxies with stellar masses M_*_ > 10^11^ *M*_⊙_ decreases dramatically, with most simulations containing zero such galaxies at *z* ≥ 5. The inferred star formation history of RUBIES-EGS-QG-1, for which star formation is quenched at *z* ≳ 5.5, therefore, implies that it would be a significant outlier at earlier times.

This indicates that the star formation and feedback recipes in the simulations do not accurately capture the formation and quenching processes of early massive galaxies. Alternatively, to reconcile the formation history of RUBIES-EGS-QG-1 with simulations requires that our estimated observed number density is substantially overestimated and that the galaxy, instead, resides in a very rare and, therefore, massive halo (*M*_halo_ ≳ 10^13^ *M*_⊙_ by *z* ≈ 5). The effects of cosmic variance may be large for the relatively small area targeted spectroscopically with JWST. Although unlikely, it is possible that the observation of RUBIES-EGS-QG-1 is an ≳3*σ* chance finding and that it, indeed, resides in a very massive halo.

**Fig. 3:**
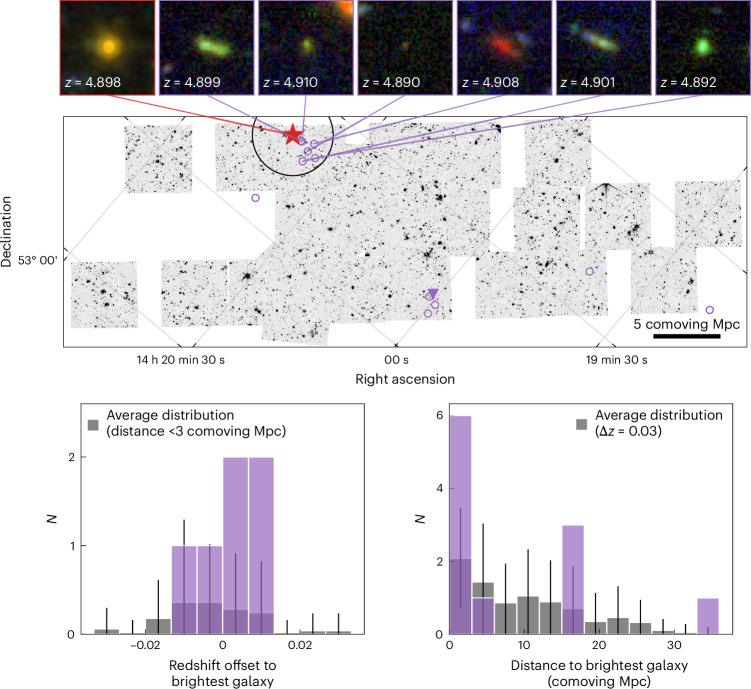
Spatial clustering of spectroscopically confirmed sources at *z* ≈ 4.90 around RUBIES-EGS-QG-1. Middle, There are 13 sources (circles) at approximately the same redshift as the quiescent galaxy (red star), six of which are nearby neighbours. The submillimetre galaxy, the brightest source among the group of four at a projected distance of 16 comoving Mpc, is indicated by a purple triangle. The background image shows the NIRCam F444W mosaic of the EGS field. Bottom, Grey histograms show the mean projected spatial clustering (within redshift ranges Δ*z* = 0.03) and redshift clustering (within apertures of radius <3 Mpc) for galaxies in the redshift range 4 < *z* < 6 with robust redshifts from JWST spectroscopy. Error bars show the standard deviation. RUBIES-EGS-QG-1 clearly resides in an overdense environment, forming the highest redshift known overdensity hosting a massive quiescent galaxy. Top, False-colour images (created from NIRCam F150W, F277W and F444W images) of RUBIES-EGS-QG-1 and its six nearby neighbours.

We examined the environment of RUBIES-EGS-QG-1 for evidence of such a large-scale overdensity by compiling all sources in the EGS with robust redshifts from JWST spectroscopy, obtained from a mixture of JWST cycle 1 and 2 programmes using the DAWN JWST Archive (DJA; Methods). We found six sources in the direct vicinity of RUBIES-EGS-QG-1 with a redshift separation Δ*z* < 0.013 and projected separation of <1 arcmin (<2 comoving Mpc) and another seven sources spread across the field at the same redshift but with larger angular separation (Fig. 3). Notably, we found a clustering of four sources at a distance of ~16 comoving Mpc from RUBIES-EGS-QG-1, of which the brightest galaxy (measured at 4 μm) is a submillimetre galaxy identified previously in SCUBA-2 data^35^. In comparison with the average density of the 4 < *z* < 6 galaxy population, we found clear evidence for an overdensity at *z* = 4.90 within an aperture of π × 3 Mpc^2^ (as also suggested in the literature^36,37^), which forms the highest-redshift overdensity containing a massive quiescent galaxy found thus far^38^. However, based on these data alone, we cannot conclusively determine whether the region, indeed, represents an extremely massive halo or whether the other seven sources are associated with the overdensity.

That the galaxy resides in an overdense environment may also point to substantial ex situ mass accretion, in addition to in situ star formation. A major merger between two approximately equally massive systems would provide a rapid accretion of stellar mass, allowing for more conventional star formation efficiencies. Moreover, major mergers have been proposed as a quenching mechanism^39^. However, equal-mass mergers between massive galaxies are exceptionally rare at *z* > 5, as the simulations predicted a number density of ≲10^−8^ Mpc^−3^ for such systems. We also did not find signatures of recent merging in the morphology of RUBIES-EGS-QG-1 (Fig. 3 and Methods).

Clearly, the rapid assembly of RUBIES-EGS-QG-1 and its early quenching requires an extreme formation scenario. In the context of recent studies that have reported candidate massive galaxies at *z* > 6 (ref. ^1^) and massive quiescent galaxies with formation times at *z* ≳ 6 (refs. ^10,40–42^), RUBIES-EGS-QG-1 stands out for its very high stellar mass, high redshift and deep Balmer absorption features, which unambiguously set its formation at *z* > 6. Theoretical models can form extremely massive galaxies at early epochs, and some also produce quiescent galaxies at *z* > 5. However, the number densities of these sources are extremely low. This may indicate that RUBIES-EGS-QG-1 lies in an extremely rare, massive halo for its redshift or the exceptional detection of a major merger between two massive galaxies. However, both of these scenarios are expected to be very rare (≲0.1 per degree squared), and the area covered by JWST spectroscopy thus far is small. The direct implication of the existence of RUBIES-EGS-QG-1 is, therefore, that the star formation and feedback prescriptions in theoretical models require revision, as the model universes cannot at present reproduce the stellar mass growth and early quenching required to match the inferred abundance of massive quiescent galaxies.

## Methods

### Spectroscopic data

The RUBIES programme (GO-4233; principal investigators (PIs) A. de Graaff and G. Brammer) is a JWST cycle 2 programme using the NIRSpec microshutter array (MSA)^43^ to observe galaxies in the CANDELS EGS and Ultra Deep Survey (UDS) extragalactic deep fields^44,45^. Specifically, RUBIES targets galaxies detected in F444W from JWST/NIRCam imaging in the Cosmic Evolution Early Release Science (CEERS; Programme 1345; PI S. Finkelstein) and Public Release Imaging for Extragalactic Research (Programme 1837; PI J. Dunlop) surveys, and it is optimized to reach high spectroscopic completeness for bright and red sources at *z* > 3. Thus far, the survey has targeted an area of approximately $100\,{{\rm{arcmin}}}^{2}$. Details of target selection and prioritization are given in ref. ^46^.

RUBIES-EGS-QG-1 (right ascension 214.9155459°, declination 52.9490183°) was observed in March 2024 as part of the observations in the EGS field. The MSA pointings were observed for 48 min each in the PRISM/CLEAR and the G395M/F290LP spectroscopic modes. Each target was observed in a 1 × 3 configuration of open microshutters with a three-point nodding pattern. The spectra were allowed to overlap on the detector in the G395M exposures as most sources have faint enough continua so as not to severely contaminate other spectra.

The NIRSpec data were reduced using the msaexp^47^ pipeline version 3, as described in ref. ^46^. Briefly, in comparison to version 2 of the pipeline described in ref. ^48^, we used updated reference files to improve flux calibration. In addition, we leveraged empty sky shutters from the RUBIES programme to derive custom bar shadow corrections, which provided a substantial improvement over the default reference files by removing strong (~10% level) unphysical discontinuities in the extracted spectrum. We also used the empty sky shutters to construct a global background subtraction for the PRISM spectra. We used local background subtraction from the nodded exposures for the G395M spectrum, as the overlapping traces on the detector in this mode made it difficult to construct a global background solution. A Gaussian profile was fitted to the two-dimensional PRISM spectra to estimate the intrinsic width and centroid of the trace. The one-dimensional spectra for both dispersers were then extracted using this Gaussian profile with an optimal weighting^49^. We scaled up the 1*σ* errors on the one-dimensional extracted spectrum by a factor of 1.7 to account for underestimated uncertainties when comparing the pixel-to-pixel variations with the msaexp-derived errors, as described in ref. ^50^. The final flux calibration of the RUBIES-EGS-QG-1 spectrum was performed by matching the continuum level in PRISM to the multi-band photometry from Hubble Space Telescope and JWST/NIRCam, as described in ‘Photometric data’.

### Photometric data

We used publicly available JWST/NIRCam imaging from the CEERS programme^51^ as well as programme GO-2234 (PI Bañados; Khusanova et al. in preparation), which combined provided eight photometry bands (F090W, F115W, F150W, F200W, F277W, F356W, F444W and F410M).

We used the reduced image mosaics from the DJA (v.7.4). All images were reduced using grizli^52^ and have a pixel scale of 0.04 arcsec pix^−1^ (see ref. ^9^ for further details of the reduction). Next, we used empirical point spread function (PSF) models to construct mosaics that were PSF-matched to the F444W mosaic, as described in ref. ^53^. We measured fluxes in circular apertures with a radius of 0.25 arcsec from the PSF-matched photometry, which was centred on the centroid position estimated by running SourceExtractor^54^ on an inverse-variance weighted stack of the F277W, F356W and F444W bands.

We show image cut-outs of the F115W and F444W NIRCam filters in Supplementary Fig. 1 and include the positions of the NIRSpec microshutters. The centroid of RUBIES-EGS-QG-1 was at the bottom of a microshutter, with some of the light falling on the bar between two microshutters. Due to the large variation in the PSF width as a function of wavelength, the slit losses introduced by the bar shadow were highly complex, the effect of which we discuss further in ‘SED modelling’. We also found a faint blue clump in the F115W image, located ~0.2 arcsec from RUBIES-EGS-QG-1. In ‘Fitting the emission lines’, we show that emission lines from this source reveal that it is a satellite with a velocity separation of ~600 km s^−1^.

### SED modelling

To measure the stellar population properties of RUBIES-EGS-QG-1, we used the Bayesian spectral energy distribution (SED) fitting code Prospector^15,55,56^ to fit non-parametric star formation histories to the NIRSpec/PRISM spectrum and the JWST/NIRCam photometry of this galaxy. We enforced a signal-to-noise ceiling of 20 on our photometric measurements to account for systematic uncertainties in the underlying stellar population models. We used flexible stellar population synthesis models^57,58^, the MILES spectral library^59^ and MIST isochrones^60,61^. We assumed a Chabrier initial mass function^62^ and fixed the model redshift to *z*_prism_ = 4.906, the PRISM spectroscopic redshift estimated with msaexp, which differs slightly from the redshift derived from the G395M spectrum due to wavelength calibration uncertainties between the NIRSpec dispersers^63^. We masked the outer edges of the spectrum to avoid uncertainties in modelling absorption from the intergalactic medium (blueward of rest frame 1,200 Å) and the absolute flux calibration at the edge of the NIRSpec CLEAR filter where we did not have photometric coverage (>5.1 μm).

Our parameterization for the fiducial star formation history was a 14-bin non-parametric model using the Prospector continuity prior, with the logarithmic ratio between neighbouring bins fitted with a Student’s *t*-distribution prior centred at 0 with a width of 0.3 and *ν* = 2 following ref. ^64^. We divided the most recent 100 Myr of star formation into three bins of widths 5, 25 and 75 Myr, respectively, to provide fine sampling of the most recent star formation history, and we filled the remaining age of the Universe with 11 linearly spaced 100 Myr bins. We assumed a two-parameter dust law with free *A*_v_ and dust index spanning [0, 2.5] and [−1, 0.4] respectively^65^, and we fixed the attenuation around young stars (*t* < 10^7^ Myr) to be twice that of the older populations. We fitted for a free logarithmically sampled stellar metallicity in the range [0.1 *Z*_⊙_, 2 *Z*_⊙_]. We performed sampling using the dynesty nested sampling package^66^.

To account for the NIRSpec/PRISM resolution, we convolved all models using the PRISM resolution curve provided in the JWST User Documentation (https://jwst-docs.stsci.edu), scaled by a multiplicative factor of 1.3 as in ref. ^67^, which approximated the line spread function (LSF) of a compact source. As RUBIES-EGS-QG-1 is very massive, we expected there to be substantial intrinsic broadening of the stellar continuum due to the random motions of stars. As such, we fitted for another free continuum velocity dispersion, with a deliberately large prior (*σ*_smooth_ = [0, 1,000] km s^−1^), which can also account for uncertainty in the precise normalization of the NIRSpec LSF. We also accounted for uncertainty in the NIRSpec flux calibration by fitting with the Prospector PolySpecModel prescription, which marginalized out a sixth-order multiplicative polynomial to rectify the observed spectrum to the model during each likelihood call. The choice to calibrate out such a high-order polynomial was motivated by the large wavelength baseline (~5 μm) of the PRISM spectrum coupled with the considerable uncertainty in the flux calibration on small scales due to the effect of differential slit losses. Because the source was partially on the bar between two shutters (Supplementary Fig. 1) and the PSF of JWST depends strongly on wavelength, the effect of the bar shadows also had a strong wavelength dependence. Our empirical bar shadow correction (‘Spectroscopic data’) derived from blank sky shutters provided only a first-order correction of this effect, as the sky emission filled the slit uniformly but the morphology of RUBIES-EGS-QG-1 followed a steep Sérsic profile (‘Size measurements’). This conservative approach used the spectrum only for sharp spectral features, such as emission or absorption lines and spectral breaks, and relied on the better-calibrated photometry to fix the shape of the SED. Note that using a lower-order polynomial (for example *n* = 1) yielded a similarly old, low-metallicity, stellar population (see also ‘Testing the star formation history’), although with a marginally lower stellar mass and more extended star formation history than our fiducial fit. However, this fit had a significantly higher *χ*^2^ value (${\chi }_{n = 1}^{2}-{\chi }_{n = 6}^{2}\approx 100$) and there were oscillatory features in the residuals, indicative of flux calibration issues.

Finally, we accounted for nebular emission in key emission lines (Lyα_1216_, [O ii]_*λλ*3727,3729_, [O iii]_*λλ*4960,5008_, Hδ_*λ*4103_, Hγ_*λ*4342_, Hβ_*λ*4864_, Hα_*λ*6564_, [N ii]_*λλ*6549,6585_ and [S ii]_*λλ*6718,6733_) using the Prospector nebular marginalization procedure, which fitted for emission lines in the residual spectrum using a least squares algorithm and incorporated those models in each likelihood call. This procedure allowed the emission lines to be produced without ascribing the emission to a specific source, which was important given the strong evidence for non-star formation sources of ionizing radiation (‘Fitting the emission lines’), although our conclusions were unchanged when we allowed for physical nebular infilling of the Balmer absorption lines. As for the stellar continuum, we convolved all emission lines with a free emission-line velocity dispersion *σ*_gas_ = [0, 1,000] km s^−1^, where we assumed that all emission lines have the same width. Supplementary Fig. 2 shows the covariant posteriors for key parameters characterizing RUBIES-EGS-QG-1 and demonstrates that the fits were well converged.

### Testing the star formation history

The central result of fitting the star formation history of RUBIES-EGS-QG-1 was the finding that the galaxy assembled its high stellar mass (~10^11^ *M*_⊙_) and ceased forming stars within only the first billion years of cosmic time, thus straining galaxy evolution models. Here, we explore how different choices for the parameterization of the star formation history or our priors could impact the inferred age of this system.

First, we examined the impact of choosing a different parameterization for the star formation history. Non-parametric models for the star formation history are commonly chosen because of their flexibility to account for a wide range of star formation history shapes, but such parameterizations systematically measure older ages with increased uncertainty^68^. As a soundness check, we performed our fiducial fit with a star formation history parameterized with the commonly used delayed *τ* (SFR ∝ *t*e^−*t*/*τ*^) with just three free parameters describing the star formation history rather than 14. We found that this parametric star formation history almost identically recovers that of the non-parametric model, measuring ${t}_{{\mathrm{form}}}=480_{-40}^{+30}$ Myr and recovering similar posteriors for the star formation rate, stellar mass, metallicity and *t*_90_ (Supplementary Fig. 3 and Supplementary Table 1), albeit with a slightly longer difference between the formation time of 10% and 90% of the total stellar mass, *t*_90_ − *t*_10_.

Second, we tested our non-parametric star formation history against the choice of prior to mitigate the concern that the inferred star formation history was prior-driven^64,69^. Instead of the flat prior assumed in our fiducial set-up, we imposed a rising star formation history prior, as expected from, for example, refs. ^70,71^, by shifting the mean logarithmic ratio of our Student’s *t* prior from 0.0 to 0.3 and rerunning our fiducial fit. We found that our measurement of *t*_form_ was largely insensitive to the imposition of this prior and that the galaxy was still fitted as ~550 Myr old, despite a slight increase in the implied star formation rate and formation time.

We found that both the fiducial fit (insensitive to the prior for the star formation history) and delayed *τ* fits converged to subsolar metallicities, with all models suggesting that the metallicity of RUBIES-EGS-QG-1 is 15–20% solar. Our measurements of metallicity were indirect, in the sense that they were sensitive to the shape of the SED rather than specific spectral features due to the low resolution of the PRISM spectrum and the relative weakness of metal-sensitive features compared to the bright A-type stars that dominate the light of RUBIES-EGS-QG-1. However, these measurements are qualitatively consistent with the finding that massive quiescent galaxies at *z* > 1.4 are metal poor relative to solar abundances^16,72^ and with comparably high-redshift massive systems^10^. On the other hand, because of a lack of very young metal-poor stars in the Milky Way, empirical model libraries suffer from calibration issues for young ages and low metallicities, which together with uncertainties in the stellar isochrones can have a strong effect on the shape of the continuum of the resulting stellar population synthesis models^73,74^. We found that fits made using the C3K theoretical stellar libraries^75^ rather than the empirical MILES libraries resulted in low-metallicity solutions within ~0.1 dex of our fiducial runs and with comparable age measurements, highlighting that the preference of our fits to these low-metallicity solutions is insensitive to the choice of model libraries. Importantly, however, these model libraries all assume solar abundance patterns. Recent results indicate that deviations in the elemental abundance patterns from the solar abundances used in the stellar population synthesis models can also lead to incorrectly inferred metallicities^16^. Given that we measured very high [N ii]/Hα, which can locally be produced only in an AGN with high metallicity^76^, we also fitted the galaxy under the assumption of solar metallicity to quantify the effect that a model mismatch would have on the star formation history we inferred.

We found that fixing the metallicity to solar affected the goodness of fit, as evidenced by the slightly worse ${{\chi }^{2}}/{{N}_{{\rm{data}}}}$ values of the fit (where *N*_data_ is the number of data points in the spectrum, Supplementary Fig. 3). However, this difference was largely driven by differences in the subtle shape of the rest ultraviolet, where the NIRSpec PRISM resolution is worst and where stellar population libraries differ substantially. As such, it was difficult to reject this solution, even if the models formally favoured low-metallicity solutions. Choosing to fix the metallicity to solar had a negligible effect on the measured stellar mass, moving the median value by ~0.05 dex, but had a considerable impact on the inferred star formation history. At solar metallicity, the observed spectrum and photometry of RUBIES-EGS-QG-1 is best described by a stellar population that formed 50% of its mass ~400 Myr later than the low-metallicity fiducial model. The fit still implies that the galaxy assembled most of its mass within the first gigayear of cosmic time at the 2*σ* level, but the measured redshift where the galaxy had assembled 90% of its mass (and can be considered to have quenched) shifted from $8.6_{-0.1}^{+0.1}$ in the fiducial model to $5.7_{-0.0}^{+0.1}$ in the fixed-metallicity model. The fixed-metallicity fit also implies a very similar burst shape to the fiducial fit. Despite the later formation time, the estimated peak star formation rate, star formation duration and star formation decline timescale (bottom panels in Supplementary Fig. 3) match closely between all models. Finally, the fixed-metallicity star formation history still implies an extremely efficient star formation history, requiring *ε* > 0.2 for the estimated number density of the source (Fig. 2).

Future deep observations that can directly measure metal-sensitive features and that are less beholden to the low resolution of the NIRSpec/PRISM may enable us to place more robust constraints on the precise age, metallicity and dust content of this system. However, despite uncertainty in the exact age of the stellar population, the observed PRISM spectrum and photometry unambiguously demonstrate that RUBIES-EGS-QG-1 is a massive quiescent galaxy (M_*_ ≈ 10^11^ *M*_⊙_) that assembled most of its mass within the first ~1 Gyr of cosmic time before rapidly quenching at *z* > 5.5, which strains current theoretical models (‘Theoretical predictions’).

### Fitting the emission lines

The G395M spectrum obtained with JWST/NIRSpec (Supplementary Fig. 4) has a higher spectral resolution (*R* ≈ 1,000–1,500) and resolves the emission-line doublets and the Hα and [N ii] complex that are blended in the PRISM spectrum. We also found several emission lines that were spatially offset from the spectrum of RUBIES-EGS-QG-1. Crucially, we found that the lines at 2.95 and 3.85 μm were present in both the PRISM and G395M two-dimensional spectra. We identified these lines as [O iii] and Hα emission from the faint blue source in the F115W image (Supplementary Fig. 1). Additionally, one emission line in the spectrum at 3.80 μm was not seen in the PRISM spectrum. This implies that it originated from a different source on the NIRSpec MSA: the two-dimensional spectra for the two sources overlapped on the detector because the G395M spectra are longer than the PRISM traces.

We performed a simultaneous fitting to the continuum and the [O iii], [N ii] and [S ii] doublets. Although the extraction kernel used to obtain the one-dimensional spectrum mitigated contamination from the satellite and spurious sources, there was still some emission from these sources. We, therefore, masked the contaminant emission line at 3.80 μm but explicitly included the [O iii] and Hα lines of the satellite source in our model. We modelled each emission line with a Gaussian line profile. The line ratio of [N ii] was fixed to 1:2.94 and that of [O iii] to 1:2.98. Given the limited signal-to-noise ratio of the emission lines, we assumed that all emission (and absorption) lines have the same velocity dispersion, which was a free parameter in the fit. We also included a Hα emission-line component to estimate the infilling of the Balmer absorption line. As the Hα emission may have a different origin from the other emission lines, we left its velocity dispersion as another free parameter. To model the continuum, we used the deconvolved median posterior model from our Prospector fitting to the PRISM spectroscopy. We fitted a first-order polynomial between this continuum and the G395M spectrum to flux calibrate the spectrum. We assumed all components (except for the satellite source) were at the same redshift.

The model was convolved with a custom LSF tailored to the morphology of RUBIES-EGS-QG-1 based on the Sérsic fit at 4 μm (ref. ^77^). We allowed for uncertainty in the LSF following the method described in ref. ^78^. To estimate the posterior distributions of the parameters, we used the emcee package to perform Markov chain Monte Carlo sampling. We adopted uniform priors for all parameters, allowing for velocity dispersions in the range *σ*_gas_ ∈ [0, 750] km s^−1^. We set a broader prior for the Hα velocity dispersion, *σ*_Hα_ ∈ [0, 1,500] km s^−1^, to test whether there was evidence for a broad-line AGN.

The emission-line fluxes are reported in Supplementary Table 2. We show the median posterior model of the combined continuum and emission-line fitting in Supplementary Fig. 4 as well as the individual emission-line and continuum components. From the fitting, we obtained a redshift of ${z}_{{\rm{spec}}}=4.897{6}_{-0.0010}^{+0.0006}$ and ionized-gas velocity dispersion of ${\sigma }_{{\rm{gas}}}=41{4}_{-64}^{+56}\,{\rm{km}}\,{{\rm{s}}}^{-1}$. The velocity dispersion of the Hα line converged to a similar value, ${\sigma }_{{\rm{H\alpha }}}=46{1}_{-150}^{+163}\,{\rm{km}}\,{{\rm{s}}}^{-1}$. The satellite source has a redshift of ${z}_{{\rm{spec}}}=4.888{5}_{-0.0009}^{+0.0009}$ and is, thus, offset by approximately 600 km s^−1^ in the rest frame.

We found very weak Hα emission, marginally detected at the 2*σ* level. We, hence, measured the emission-line ratio log([N ii]_*λ*6585_/Hα) $=0.5{0}_{-0.25}^{+0.34}$. Although the Hβ line falls outside the wavelength range covered by the G395M spectrum, we obtained a lower limit on the Hβ emission-line flux by assuming case B recombination (Hα/Hβ = 2.86). In this case, the lower limit on the ratio $\log$([O iii]_*λ*5008_/Hβ) = $0.5{0}_{-0.26}^{+0.31}$. These line ratios indicate that the line emission in RUBIES-EGS-QG-1 did not originate from star formation^12^. Although shocked gas also results in high [N ii]/Hα line ratios, the ratio inferred from the spectrum and lower limit on the [O iii]/Hβ ratio are most probably consistent with ionization by an AGN^13,79,80^.

We used the ionized-gas velocity dispersion to estimate the dynamical mass using the methodology presented by ref. ^81^ so that we could relate the gas kinematics to the gravitational potential for compact quiescent galaxies. This assumed that the gas forms a rotating disk, with a rotational velocity that is related to the integrated gas velocity dispersion and the inclination (*i*) of the disk: ${v}_{{\rm{rot}}}={\sigma }_{{\rm{gas}}}/(\alpha \sin (i))$, where *α* ≈ 0.8. Based on our morphological modelling (‘Size measurements’), we found that the projected axis ratio *q* ≈ 0.85. Assuming an intrinsic disk thickness of *q*_0_ = 0.2, this implies *i* = 32.5°. The dynamical mass was computed as ${M}_{{\rm{dyn}}}=2{v}_{{\rm{rot}}}^{2}{r}_{{\rm{e}}}/G$, where *r*_e_ is the inferred half-light radius (‘Size measurements’) and *G* the gravitational constant. With *r*_e_ = 0.55 ± 0.01 kpc, we found ${M}_{{\rm{dyn}}}=2.{7}_{-0.8}^{+0.7}\times 1{0}^{11}\,{{{M}}}_{\odot }$, a factor ~3 higher than the measured stellar mass. Note that the gas kinematics may be a biased tracer of the gravitational potential, as the observed stellar and ionized-gas kinematics for a large sample of galaxies at *z* ≈ 1 have been shown to agree well on average^82^ but with a systematic offset of approximately 0.2 dex for *σ*_gas_ ≈ 400 km s^−1^. This may imply that the dynamical mass is overestimated by 0.4 dex (a factor of 2.5, *M*_dyn_ ≈ 1.1 × 10^11^ *M*_⊙_), which is consistent with the estimated stellar mass.

### Size measurements

We measured the effective radius (*r*_e_) of RUBIES-EGS-QG-1 as a function of wavelength using the GALFIT^83,84^ single-component Sérsic fitting methods from ref. ^85^ in all available filters (F115W, F150W, F200W, F277W, F356W, F410M and F444W), corresponding to rest-frame wavelengths in the range ~2,000 to 7,500 Å.

All filters were processed following the procedure described in ref. ^86^. The parameters were constrained in the model fits as follows. The magnitude could range ±3 mag from the photometric catalogue value, the radius could vary over 0.01 < *r*_e_ < 400 pixels, the Sérsic index could range from *n* = 0.2 to 10, and axis ratio could range from *q* = 0.0001 (flat) to *q* = 1 (round).

Next, sizes were corrected to account for residual flux using the methods of ref. ^87^. The growth curve from the best-fitting Sérsic model (deconvolved from the PSF) was added to the GALFIT residual growth curve. We extrapolated the combined growth curve using the Sérsic model alone when the annular signal-to-noise ratio <3 for the science image. The corrected radius was then defined as the radius where the residual plus unconvolved Sérsic growth curve reached 50%.

We found that RUBIES-EGS-QG-1 is remarkably compact, although we unambiguously resolved this galaxy in F356W, F410M and F444W. The F444W fit is shown in Supplementary Fig. 5, which suggests that it has a very compact profile (*n* = 8.80 ± 0.14) and a corrected half-light radius *r*_e_ = 0.55 ± 0.01 kpc (consistent with ref. ^88^). The F356W and F410M fits gave similar Sérsic indices (8.77 and 8.15, respectively) and corrected radii (0.50 and 0.58 kpc), indicating a flat colour gradient in the rest-frame optical (3,400 to 7,500 Å). Given the rapid assembly and quenching of RUBIES-EGS-QG-1, it was not surprising to find a flat colour gradient. See, for example, ref. ^89^. The shorter wavelength images (rest frame <3,000 Å) are very compact, and we had trouble converging them to accurate fits without hitting the *n* = 10 upper limit.

### Environment at *z* = 4.9

Recent studies have speculated about the existence of an overdensity at *z* ≈ 5 in the EGS field, based on a clustering of photometric redshifts^37^ and a sample of four spectroscopic redshifts at *z* ≈ 4.90 obtained with JWST/NIRSpec^36^. With the addition of RUBIES-EGS-QG-1, this gives a sample of five spectroscopic redshifts.

To search for further evidence of a large-scale overdensity, we leveraged all available spectroscopic redshifts from JWST/NIRSpec in the EGS field. These spectra come from different JWST cycle 1 and 2 programmes: the CEERS programme, a director’s discretionary time programme (ID 2750; PI Arrabal Haro) and the RUBIES programme. All data from the DJA were reduced using the msaexp pipeline^47^, in the same manner as described in ‘Spectroscopic data’. Redshifts were obtained from the spectra using *χ*^2^ minimization template fitting with msaexp and visually inspected to evaluate the quality of the redshifts. The redshifts were predominantly derived from PRISM spectroscopy, although approximately half of the sources in CEERS were observed with the medium-resolution gratings only. We began by compiling all sources with robust redshifts (grade 3 from visual inspection) in the range 4 < *z* < 6 from the DJA, which resulted in 193 sources from RUBIES, 123 from CEERS and another 20 from the director’s discretionary time programme. Of these 336 sources, 289 were detected in NIRCam imaging, with the remainder falling outside the NIRCam footprint.

We selected galaxies within Δ*z* = ±0.0135 (Δ*v* = 4,000 km s^−1^) from the redshift of RUBIES-EGS-QG-1, chosen based on the typical redshift ranges used to search for overdensities (from the compilation in ref. ^90^), which is sufficiently large to account for systematic uncertainties in the wavelength calibration of NIRSpec^63^. We found 13 sources in this velocity range around RUBIES-EGS-QG-1 (Supplementary Fig. 6 and Supplementary Table 3), four of which were published previously. Of these 13 sources, six have a close angular separation to RUBIES-EGS-QG-1 of <1 arcmin, corresponding to <400 kpc or <2 comoving Mpc. We identified another clustering of four sources at a distance of ~7 arcmin (~16 comoving Mpc) from RUBIES-EGS-QG-1. Interestingly, the brightest source in the F444W imaging among these four (RUBIES-EGS-14295) is a submillimetre galaxy identified from SCUBA-2 850 μm data (S2CLS-EGS-850.061)^35^, which implies a high star formation rate. The remaining three sources were scattered across the field at varying distances.

The typical NIRSpec MSA can target approximately ~200 sources simultaneously, which generally leads to a complex selection function such that high-priority targets have a high probability of being allocated a shutter whereas an individual galaxy in the broader population has a low probability of being observed^43,63,91^. As a result, the spectroscopic completeness is probably low at intermediate redshift, as the science objective of moist programmes focuses on galaxies at *z* > 6. Because of this incompleteness, it was difficult to quantify the overdensity of the spectroscopic targets at the redshift of RUBIES-EGS-QG-1 to estimate its halo mass. However, we used the 4 < *z* < 6 galaxy population with robust spectroscopy to assess qualitatively whether RUBIES-EGS-QG-1 is probably part of an overdensity. Under the assumption that sources in this redshift range are targeted with approximately equal probability, we estimated the typical clustering of galaxies in redshift space at the projected distance.

To assess the expected clustering in redshift space, we selected the 50 brightest sources in F444W NIRCam imaging among the 336 spectroscopic targets. We then searched for sources within a radius of 3 comoving Mpc, approximately three times the virial radius of a moderately sized cluster at *z* = 0 (*M*_halo_ ≈ 10^14.5^ *M*_⊙_), and we computed the redshift separation between each source and the brightest galaxy. The corresponding distribution in the redshift space peaked at Δ*z* = 0 but was very broad (Fig. 3). Within the range Δ*z* = 0.027, we found that the overdensity around RUBIES-EGS-QG-1 is significantly (3.1*σ*) above this background level. When we used a larger aperture, we found only marginal detections of 2.2*σ* (5 Mpc), 1.5*σ* (10 Mpc) and 1.6*σ* (20 Mpc).

Similarly, we selected all sources in narrow redshift windows of Δ*z* = 0.027, stepping between *z* = 4.0 and 6.0, and we measured the angular separation with respect to the brightest source in the redshift slice. We again found a broad distribution in the projected distance, which we used to estimate the significance of the clustering of sources around RUBIES-EGS-QG-1. Within a radius of 3 Mpc, the neighbours of RUBIES-EGS-QG-1 were 2.8*σ* above the expected level. As before, this decreased when searching within larger apertures: 2.0*σ* within 5 Mpc, 1.0*σ* within 10 Mpc and 1.44*σ* within 20 Mpc.

We concluded that RUBIES-EGS-QG-1, indeed, resides in an overdense environment, at least within an aperture of 3 comoving Mpc. At present, this is the highest-redshift overdensity containing a massive quiescent galaxy^38^. However, based on these findings we could not determine whether RUBIES-EGS-QG-1 resides in a massive halo or in the extremely massive halo needed to reconcile the observed number density of the galaxy with expectations from galaxy formation simulations. Further spectroscopic follow-up observations will be critical for performing the thorough analysis of the spectroscopic completeness needed to obtain a robust estimate of the halo mass at the observed redshift and the projected evolution to the present day.

### Theoretical predictions

We compared the existence and properties of RUBIES-EGS-QG-1 with five different large-volume simulations from recent literature, all of which simulated galaxy formation and evolution within the Λ cold dark matter (CDM) cosmological model. First, we evaluated the number density of massive quiescent galaxies in the different models:

- The First Light and Reionisation Epoch Simulations (FLARES)^92,93^ uses the EAGLE model^94,95^ to perform hydrodynamical zoom simulations of regions in a volume of 3.2 comoving Gpc^3^, thereby probing rare haloes that would not appear in the EAGLE simulation. Using the measurements of ref. ^30^ based on the combination of FLARES and EAGLE, the predicted surface density of quiescent galaxies at *z* ≈ 4.5–5 and apparent magnitude like that of RUBIES-EGS-QG-1 (F200W < 24.5) is approximately 0.1–1 per degree squared or *n* ≈ 1 to 10 × 10^−8^ Mpc^−3^.
- In the cosmological hydrodynamical simulation TNG300 of the IllustrisTNG project^96–101^, which simulated a comoving volume of 302 Mpc^3^ using the IllustrisTNG model, the population of massive quiescent galaxies appears in the simulation only at *z* ≈ 4.2 (ref. ^32^). At *z* = 5, we found a single system in the simulation that lies within the 3*σ* contours of the stellar mass and star formation rate of RUBIES-EGS-QG-1 (as measured within an aperture of twice the three-dimensional stellar half-mass radius), corresponding to a comoving number density of *n* = 3 × 10^−8^ Mpc^−3^.
- In the cosmological hydrodynamical simulation Magneticum Pathfinder with a comoving volume of 180 Mpc^3^, lower-mass quiescent galaxies are present with number densities consistent with observations^31^, but no systems were reported at M_*_ ≈ 10^11^ *M*_⊙_ at *z* ≈ 5, setting an upper limit on the number density of *n* < 1 × 10^−7^ Mpc^−3^.
- The latest version of the semi-analytic Galaxy Evolution and Assembly (GAEA) model^33,102–104^ was run on the Millennium Simulation^105^ with a box length of 500 Mpc/*h* (where *h* = 0.73). Within this large volume, there were 14 quiescent galaxies with $\log ({M}_{* }/{{{M}}}_{\odot }) > 10.9$ at *z* ≈ 4.9, that is *n* = 4 × 10^−8^ Mpc^3^.
- The semi-analytic model SHARK v.2.0 (ref. ^34^) was run for the SURFS^106^ dark matter-only simulation volume with a box length of 210 Mpc/*h* where *h* = 0.6751. Following the methodology of ref. ^34^, which includes an estimate of the observational uncertainty of 0.25 dex on the inferred number density, the number density of massive ($\log ({M}_{* }/{{{M}}}_{\odot }) > 10.9$) quiescent galaxies *n* = 2 × 10^−8^ Mpc^3^ at *z* = 5.

In summary, the different models all predict the existence of massive quiescent galaxies at high redshifts but with very low number densities at the redshift of RUBIES-EGS-QG-1 and with a typical value of *n* ≈ 5 × 10^−8^ Mpc^−3^ across the simulations. With an estimated observed number density of *n* ≈ 4 × 10^−6^ Mpc^−3^, RUBIES-EGS-QG-1 is, therefore, an outlier at the 2*σ* level at *z* ≈ 5, if we assume the theoretical model predictions to be accurate. It also implies that, instead of residing in a halo with *M*_halo_ ≈ 10^12^ *M*_⊙_, the halo mass of RUBIES-EGS-QG-1 would be among the most massive haloes at *z* = 5, as *M*_halo_ ≈ 10^12.5–13^ *M*_⊙_ in the simulations.

However, at higher redshift, the tension increases. Even for the solar-metallicity model, RUBIES-EGS-QG-1 formed its stars and quenched at *z* ≳ 5.5. The FLARES simulation predicted an extremely low source density of *n* < 1 × 10^−8^ Mpc^−3^ at *z* ≥ 5.5 (≲0.1 per degree squared), and the GAEA simulations predicted similarly low numbers (*n* = 6 × 10^−9^ Mpc^−3^ at *z* ≈ 6). Both reported zero such galaxies at *z* ≈ 7, which implies that *n*(*z* = 7) ≲ 1 × 10^−9^ Mpc^−3^. The relatively smaller volumes (TNG300 and Magneticum Pathfinder) do not seem to contain any quiescent galaxies more massive than M_*_ > 10^10.9^ *M*_⊙_ at *z* ≥ 5 and do not contain any such massive galaxies at *z* > 6, even when considering star-forming galaxies. Therefore, if we also account for the unusual formation history of RUBIES-EGS-QG-1, this implies that the probability of finding a source like RUBIES-EGS-QG-1 within the small volume of the NIRCam imaging explored for the RUBIES survey thus far is approximately 0.03% (an outlier at the >2.8*σ* level), which strains current theoretical models.

Finally, we used the GAEA and TNG300 simulations to explore the possibility that RUBIES-EGS-QG-1 formed through a rapid, major merger of two moderately massive galaxies. We estimated a merger timescale of ~200–300 Myr (ref. ^107^). Because we did not find any signatures of a recent merger in the NIRCam imaging, the merger must have occurred at *z* > 6. We, therefore, searched for the nearest massive neighbour of galaxies with $\log ({M}_{* }/{{{M}}}_{\odot }) > 10.3$ in the simulations at *z* ≳ 6. For GAEA, we found two massive pairs that are separated by <0.5 comoving Mpc at *z* = 6.2 and six such pairs at *z* = 5.7, corresponding to a number density of 6 to 20 × 10^−9^ Mpc^−3^. In TNG300, we found a single pair of massive galaxies at *z* = 6.0, which implies a number density for such equal-mass mergers of 3 × 10^−8^ Mpc^−3^. However, when tracing their merger history, we found that the pair did not merge until *z* = 5.0, which is inconsistent with the lack of merger signatures in the morphology of RUBIES-EGS-QG-1. These low number densities are, therefore, upper limits on the expected rate of equal-mass mergers of massive galaxies at high redshift, and they indicate that the formation of RUBIES-EGS-QG-1 through such a scenario is expected to be extremely rare.

### NOEMA non-detection

RUBIES-EGS-QG-1 was also covered by NOEMA observations as part of project W20CK (PIs Buat and Zavala), which was originally designed to target dusty star-forming galaxy candidates at *z* > 3 (see ref. ^108^ for further details and the data reduction process). These observations allowed us to derive a 3*σ* upper limit at 1.1 mm of <1 mJy. Assuming a typical SED (a modified black-body function with a dust temperature of *T*_D_ = 35 K and a dust emissivity index of *β* = 1.8) at *z* = 4.9, this flux density upper limit corresponds to an infrared luminosity of <8 × 10^11^ *L*_⊙_. Assuming the calibration between star formation rate and infrared luminosity of ref. ^109^, this implies a dust-obscured star formation rate of <120 *M*_⊙_ yr^−1^.

## Supplementary information

Supplementary Information Supplementary Figs. 1–6 and Tables 1–3.

## Data Availability

The unprocessed JWST data are available through the Mikulski Archive for Space Telescopes. Reduced data from JWST underlying this work are publicly available in the DJA (https://dawn-cph.github.io/dja/), as described in refs. ^9,46^.

## Code availability

All software packages used in this work are publicly available from GitHub: grizli^52^, msafit^77^, msaexp^47^ and Prospector^15,55,56^. The GALFIT software can be found on https://users.obs.carnegiescience.edu/peng/work/galfit/galfit.html.
