# Supplementary material for: Efficient formation of a massive quiescent galaxy at redshift 4.9
Source: Nat Astron. 2024 Nov 28;9(2):280–92. doi: 10.1038/s41550-024-02424-3 (PMC11842275; doi:10.1038/s41550-024-02424-3)
Supplement: Supplementary file 1 — Supplementary Figs. 1–6 and Tables 1–3. [file 41550_2024_2424_MOESM1_ESM.pdf]

nature astronomy

Supplementary information https://doi.org/10.1038/s41550-024-02424-3

Efficient formation of a massive quiescent galaxy at redshift 4.9

In the format provided by the authors and unedited

Supplementary Information

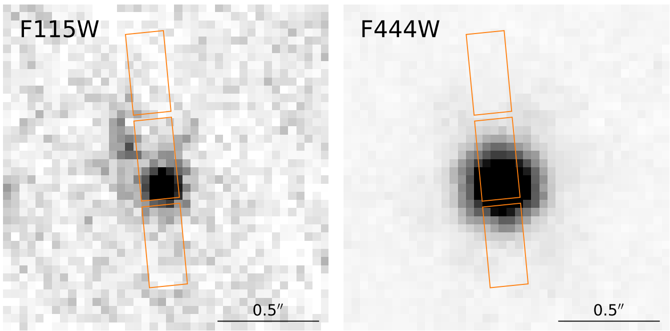

Supplementary Figure 1 NIRCam F115W and F444W image cutouts of RUBIES-EGS-QG-1. Orange lines show the location of the NIRSpec microshutters. We also identify a faint blue clump north-east of RUBIES-EGS-QG-1, which is a satellite system at approximately the same redshift (see Methods Section 5).

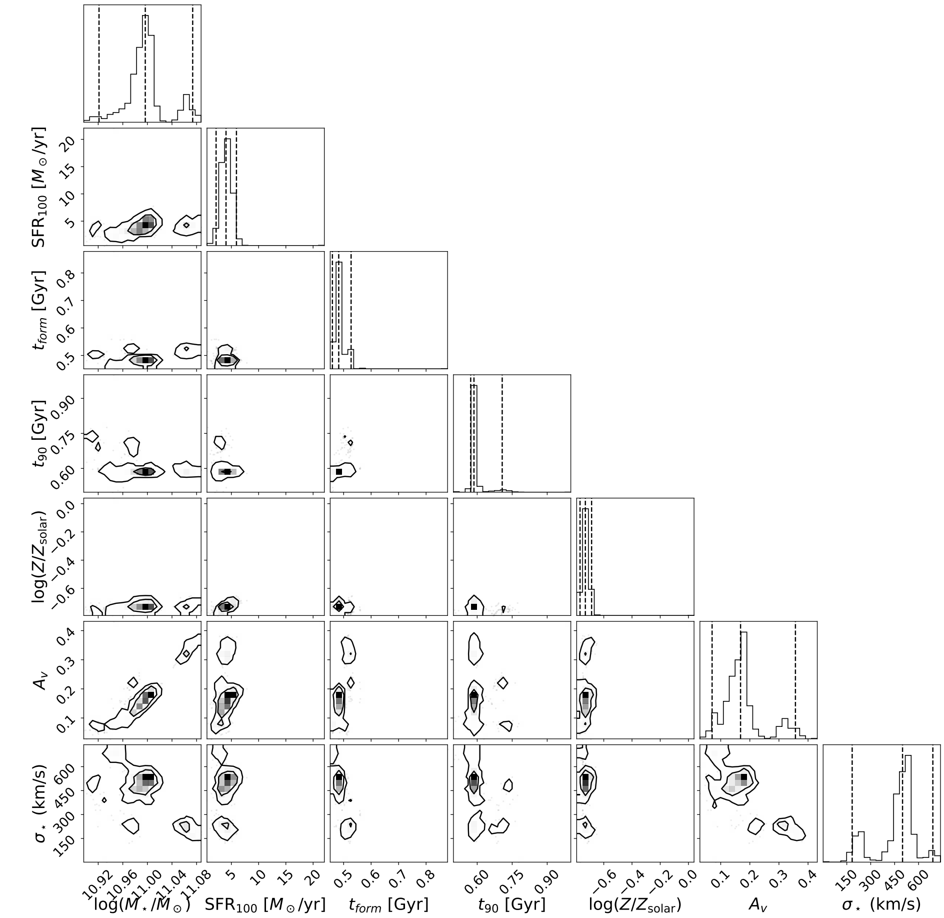

Supplementary Figure 2 Covariant posterior distributions of the fiducial stellar population model. We show a selected set of parameters of the fiducial fit with `Prospector`, focusing on the physical properties that are central to this paper. Contours bound the 68% and 95% of the likelihood and dashed lines capture the 95% confidence interval on the marginalized posteriors.

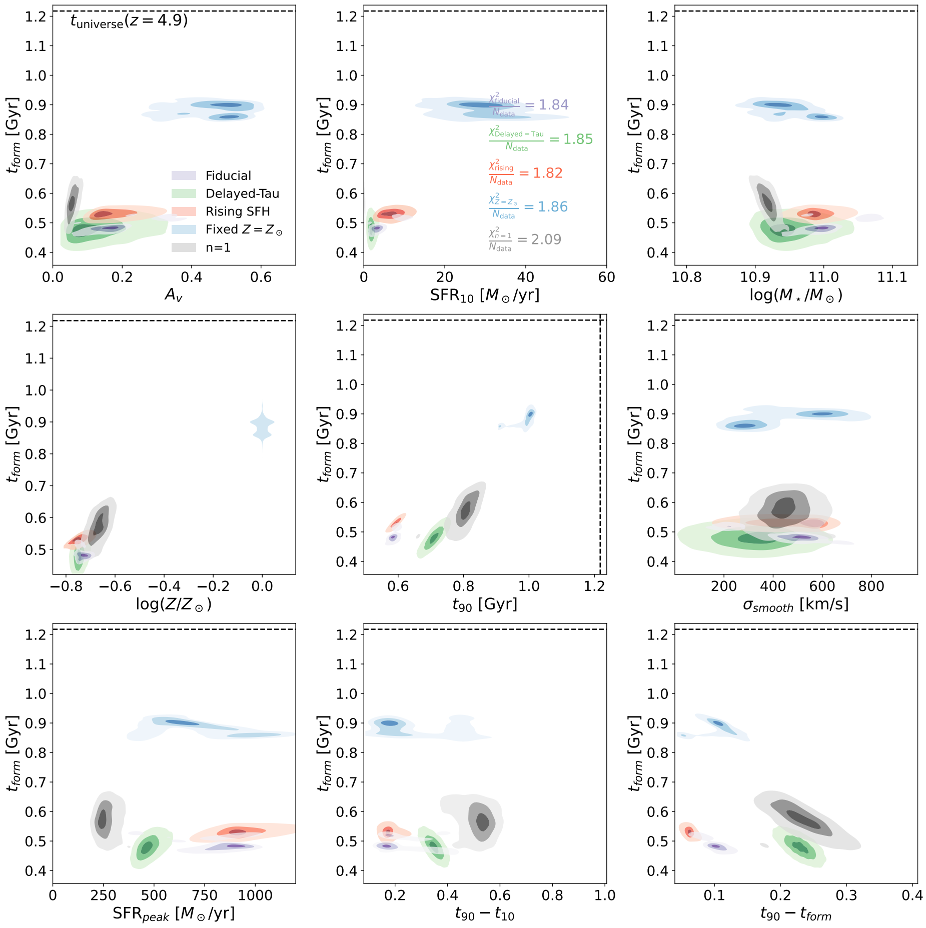

Supplementary Figure 3 Posterior distributions of the different stellar populations models. For the fiducial (purple), delayed-tau (green), our rising SFH prior (red), fixed $Z = Z_\odot$ (blue), and n=1 polynomial (grey) models, we show the age of the universe when 50% of the galaxy's mass formed, $t_{\rm form}$, against the covariant parameters of A_V (top left), star formation rate (top center), stellar mass (top right), metallicity (middle left), t_{90}, (the age of the universe when 90% of the galaxy's mass formed, middle center), $\sigma_{\rm smooth}$ (middle right), $\mathrm{SFR}_{\rm peak}$ (bottom left), star formation duration timescale ($t_{90} - t_{10}$, bottom middle), and star formation decline timescale ($t_{90} - t_{\rm form}$, bottom right). Black dashed lines indicate the age of the universe at the time of observation. The fiducial fit, the rising SFH prior fit, and the delayed-tau fits generally agree in reaching old, low-SFR, low-dust, metal-poor solutions. However, fits where we require that the average stellar metallicity to be solar result in a later-forming, more star forming, and dustier galaxy. In the bottom row, we highlight that even when metallicity is fixed to solar, the constraints on the peak star formation rate and the need for a rapid period of intense star formation are consistent with the earlier-forming fiducial model.

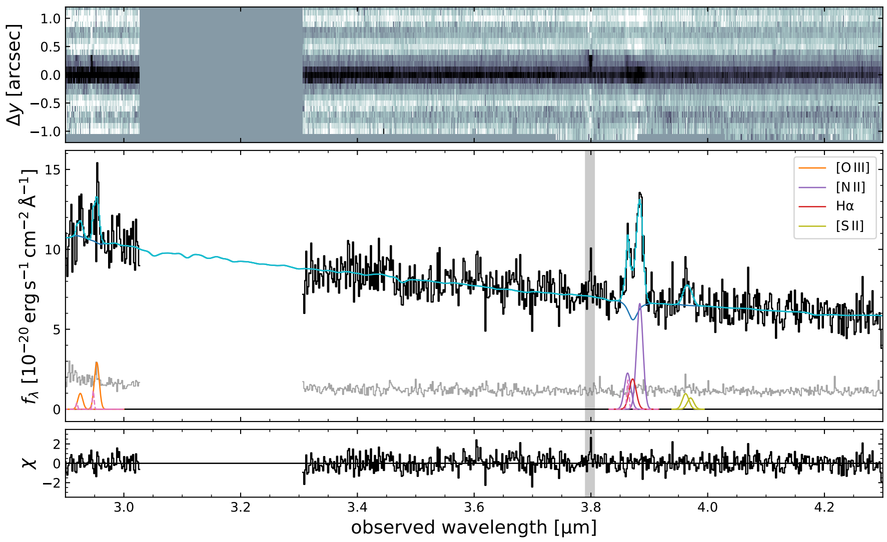

Supplementary Figure 4 JWST/NIRSpec medium-resolution spectrum of RUBIES-EGS-QG-1 , resolving the [O III], [N II], and [S II] doublets, and the Hα line. The 1σ uncertainties are show in gray. The shaded region marks wavelengths that were masked in the fitting, as this emission originates from a source in a different slit in the MSA. The dark blue line shows the median posterior model of the stellar continuum from `Prospector` . Colored lines show the median posterior models of the emission lines, and the cyan line shows the combined continuum and emission line model. Emission lines originating from a satellite source in the same microshutter, apparent from their spatial offset in the 2D spectrum, are shown with pink dashed lines.

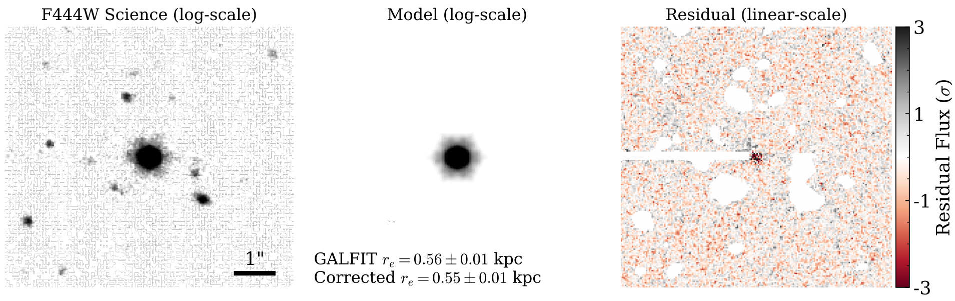

Supplementary Figure 5 Morphological fitting to NIRCam imaging of RUBIES-EGS-QG-1 . We show the original image (left), best-fit model (middle) and residual image (right); white areas in the residual image indicate pixels that were masked in the fitting. The Sérsic profile fitting for the F444W image reveals a compact light distribution ($n \approx 9$, $r_\mathrm{e} \approx 0.55$ kpc).

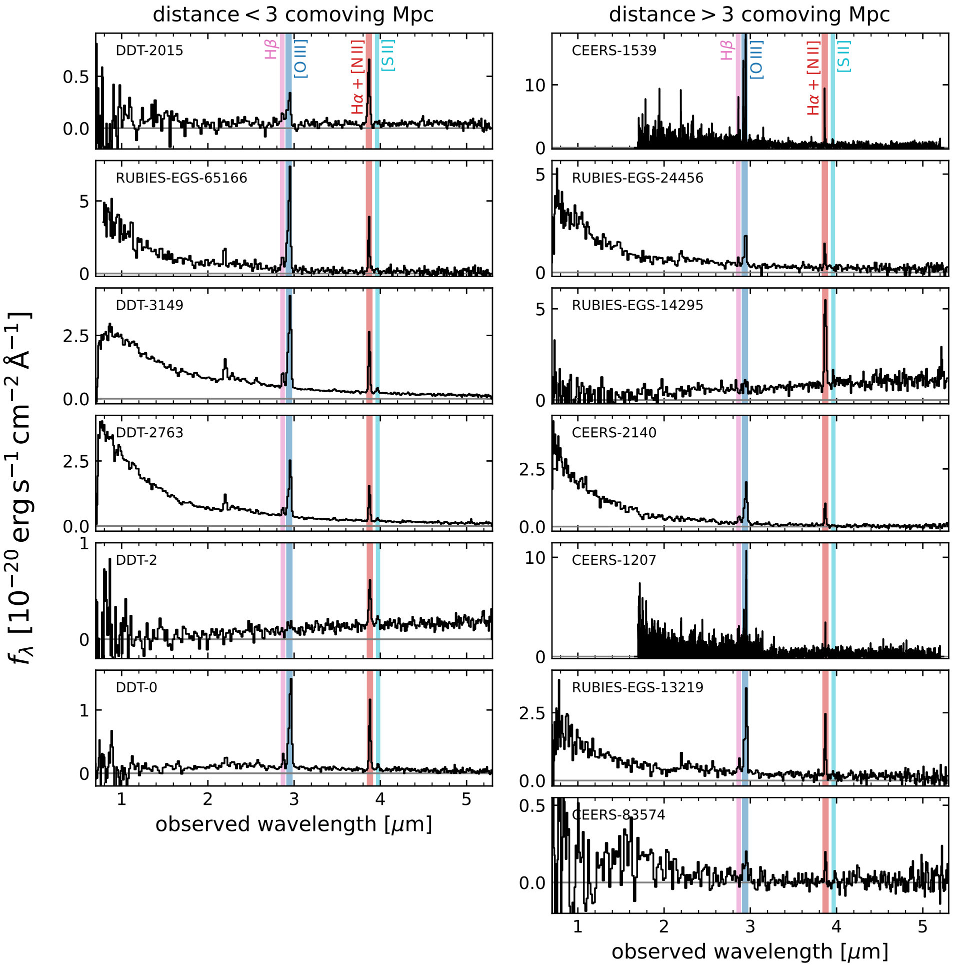

Supplementary Figure 6 JWST/NIRSpec spectra of 13 sources with a redshift $z \approx 4.90$. The left column shows objects that have a close projected separation to RUBIES-EGS-QG-1, in order of increasing redshift. The right column shows sources in the EGS that are at a larger distance from RUBIES-EGS-QG-1.

Supplementary Table 1 The resultant median and 1σ confidence intervals on a number of key galaxy parameters.

	Fiducial	Delayed-Tau	Rising	$Z = Z_\odot$	n=1
$\log(M_\star/M_\odot)$	$11.0\pm^{0.02}_{0.02}$	$10.95\pm^{0.04}_{0.03}$	$10.99\pm^{0.03}_{0.02}$	$10.94\pm^{0.05}_{0.03}$	$10.92\pm^{0.01}_{0.01}$
SFR$_{10}$ [$M_\odot$/yr]	$3.4\pm^{3.3}_{1.0}$	$2.0\pm^{0.9}_{0.6}$	$7.2\pm^{3.0}_{2.5}$	$28.8\pm^{7.8}_{8.0}$	$0.0\pm^{0.2}_{0.0}$
SFR$_{100}$ [$M_\odot$/yr]	$4.0\pm^{1.0}_{0.9}$	$2.9\pm^{1.3}_{0.9}$	$7.2\pm^{3.0}_{2.5}$	$27.4\pm^{6.4}_{15.4}$	$0.4\pm^{0.8}_{0.4}$
SFR$_{peak}$ [$M_\odot$/yr]	$870\pm^{70}_{140}$	$470\pm^{60}_{40}$	$910\pm^{140}_{100}$	$660\pm^{270}_{160}$	$260\pm^{60}_{30}$
t_{form} [Myr]	$480\pm^{30}_{10}$	$480\pm^{30}_{40}$	$530\pm^{10}_{10}$	$890\pm^{20}_{30}$	$570\pm^{50}_{50}$
z_{form}	$10.0\pm^{0.2}_{0.4}$	$9.9\pm^{0.6}_{0.4}$	$9.3\pm^{0.1}_{0.1}$	$6.3\pm^{0.2}_{0.1}$	$8.8\pm^{0.7}_{0.5}$
t_{90} [Myr]	$590\pm^{10}_{0}$	$710\pm^{20}_{30}$	$590\pm^{0}_{0}$	$1000\pm^{10}_{30}$	$800\pm^{30}_{30}$
z_{90}	$8.6\pm^{0.1}_{0.1}$	$7.4\pm^{0.2}_{0.2}$	$8.5\pm^{0.0}_{0.0}$	$5.7\pm^{0.1}_{0.0}$	$6.8\pm^{0.2}_{0.2}$
$t_{90} - t_{form}$ [Myr]	$100\pm^{10}_{10}$	$230\pm^{20}_{20}$	$60\pm^{10}_{10}$	$110\pm^{20}_{20}$	$230\pm^{40}_{30}$
$t_{90} - t_{10}$ [Myr]	$180\pm^{170}_{10}$	$350\pm^{30}_{30}$	$180\pm^{20}_{10}$	$190\pm^{270}_{10}$	$520\pm^{40}_{70}$
$A_{\rm V}$ [mag]	$0.17\pm^{0.05}_{0.05}$	$0.13\pm^{0.11}_{0.06}$	$0.18\pm^{0.08}_{0.05}$	$0.48\pm^{0.06}_{0.13}$	$0.06\pm^{0.02}_{0.01}$
$\log(Z/Z_\odot)$	$-0.73\pm^{0.02}_{0.02}$	$-0.75\pm^{0.02}_{0.02}$	$-0.74\pm^{0.02}_{0.02}$	0	$-0.67\pm^{0.04}_{0.04}$

[1]SFR averaged over the 10 Myr over observation

[2]SFR averaged over the 100 Myr over observation

[3]The age of the universe when 50% of the galaxy's mass formed

[4]The age of the universe when 90% of the galaxy's mass formed

[5]$A_{\rm V}$ surrounding $t > 10$ Myr stars. Our models assume $A_{\rm V}$ is doubled around $t < 10$ Myr stars.

Supplementary Table 2 Emission line fluxes of RUBIES-EGS-QG-1 measured from the G395M spectrum.

	flux [10^{-18} erg s^{-1} cm^{-2}]
[O III]$_{\lambda 4960}$	$1.04^{+0.31}_{-0.28}$
[O III]$_{\lambda 5008}$	$3.09^{+0.92}_{-0.83}$
[N II]$_{\lambda 6549}$	$3.09^{+0.46}_{-0.51}$
Hα	$2.87^{+1.45}_{-1.43}$
[N II]$_{\lambda 6585}$	$9.09^{+1.36}_{-1.49}$
[S II]$_{\lambda 6718}$	$1.36^{+0.91}_{-0.55}$
[S II]$_{\lambda 6733}$	$0.88^{+0.60}_{-0.54}$

Supplementary Table 3 Spectroscopically-confirmed sources in the EGS field at the redshift of RUBIES-EGS-QG-1 .

ID	R.A.	Dec.	z
CEERS-1207[1]	214.960005	52.831171	4.8957
CEERS-1539[1]	214.980078	52.942659	4.8840
CEERS-2140[1]	214.796009	52.715878	4.8927
CEERS-83574	214.949862	52.831306	4.8980
DDT-0[2]	214.914550	52.943023	4.9098
DDT-2[2,3]	214.909113	52.937204	4.9080
DDT-2015	214.917995	52.937245	4.8903
DDT-2763[2]	214.927789	52.935859	4.9009
DDT-3149[2]	214.914917	52.943621	4.8991
RUBIES-EGS-13219	214.947589	52.836578	4.8966
RUBIES-EGS-14295	214.943835	52.835816	4.8925
RUBIES-EGS-24456	214.838546	52.778963	4.8856
RUBIES-EGS-65166	214.918350	52.931829	4.8919

[1]Published in [92]

[2]Published in [36]

[3]CEERS-DSFG-1 of [93]
